# Pathogenesis-Related Genes of PR1, PR2, PR4, and PR5 Families Are Involved in the Response to *Fusarium* Infection in Garlic (*Allium sativum* L.)

**DOI:** 10.3390/ijms22136688

**Published:** 2021-06-22

**Authors:** Olga K. Anisimova, Anna V. Shchennikova, Elena Z. Kochieva, Mikhail A. Filyushin

**Affiliations:** Federal Research Centre “Fundamentals of Biotechnology” of the Russian Academy of Sciences, 119071 Moscow, Russia; lelikanis@yandex.ru (O.K.A.); shchennikova@yandex.ru (A.V.S.); ekochieva@yandex.ru (E.Z.K.)

**Keywords:** garlic *Allium sativum* L., pathogenesis-related proteins, biotic stress, *Fusarium* spp., gene structure, gene expression

## Abstract

Plants of the genus *Allium* developed a diversity of defense mechanisms against pathogenic fungi of the genus *Fusarium*, including transcriptional activation of pathogenesis-related (PR) genes. However, the information on the regulation of PR factors in garlic (*Allium sativum* L.) is limited. In the present study, we identified *AsPR* genes putatively encoding PR1, PR2, PR4, and PR5 proteins in *A. sativum* cv. Ershuizao, which may be involved in the defense against *Fusarium* infection. The promoters of the *AsPR1–5* genes contained jasmonic acid-, salicylic acid-, gibberellin-, abscisic acid-, auxin-, ethylene-, and stress-responsive elements associated with the response to plant parasites. The expression of *AsPR1c*, *d*, *g*, *k, AsPR2b*, *AsPR5a, c* (in roots), and *AsPR4a(c)*, *b,* and *AsPR2c* (in stems and cloves) significantly differed between garlic cultivars resistant and susceptible to *Fusarium* rot, suggesting that it could define the PR protein-mediated protection against *Fusarium* infection in garlic. Our results provide insights into the role of PR factors in *A. sativum* and may be useful for breeding programs to increase the resistance of *Allium* crops to *Fusarium* infections.

## 1. Introduction

Plants constantly exposed to various pathogens use complex defense mechanisms developed during evolution [1,2,3]. One of the most harmful pathogens is fungi, which developed many sophisticated mechanisms to penetrate and colonize host cells, including hydrolysis of the plant cell wall with pectinases, cellulases, and proteases [4]. The plant cell wall, containing cutin, wax, and lignin, represents the first line of defense against invading pathogens [2]. If it is broken, the plant immune system, which is the second line of defense, is activated through plant receptors that perceive pathogen-associated molecular patterns, including flagellins and components of the fungal cell wall, such as lipopolysaccharides, chitin, and branched β-glucans [5], as well as through the mobilization of resistance-related proteins that recognize pathogen effectors [6]. The result is the induction of the plant hypersensitive response, including the formation of reactive oxygen species (ROS), the induction of salicylic acid (SA) and jasmonic acid (JA) signaling, and the upregulation of pathogenesis-related (PR) genes [3]. The SA pathway, triggered mainly by biotrophic pathogens, is involved in systemic acquired resistance (SAR), which induces the expression of the *PR1*, *PR2*, and *PR5* genes and prevents the spread of infection to healthy tissues [2], whereas the JA pathway, mainly activated by necrotrophic pathogens, provides local acquired resistance (LAR) through the upregulation of *PR3*, *PR4*, and *PR12* genes [2].

The PR factors are thermostable, protease-resistant proteins of ~5–43 kDa which are expressed in all plant organs; in the leaves, they constitute about 5–10% of the total protein [7,8]. Currently, 17 PR families differing in structure, mechanisms of action, and specificity to parasites (fungi, bacteria, viruses, insects, or nematodes) are known [9,10]. Among them, PR1–5, PR12, and PR17, such as CAP-domain proteins (pfam00188; PR1), β-1,3-glucanases (PR2), chitinases (PR3), Barwin-domain proteins (PR4), thaumatin-like (PR5), and NtPRp27-like (PR17) proteins, as well as antimicrobial peptides, including defensins (PR12), were shown to be involved in the response to fungal attack [2,7,10,11].

*Fusarium* pathogens, which are widely spread in the soils of almost all climate zones, vary in nutrition type and cause two of the most destructive fungal diseases, *Fusarium* basal rot (FBR) and *Fusarium* wilt (FW), in diverse crop and wild plant species [12,13,14]. Furthermore, they produce multiple mycotoxins that are harmful to humans [15]. Therefore, the mechanisms of protection against *Fusarium* invasion have been extensively studied both in model plants [16] and in crops such as potato [17,18], tomato [19], and cereals [20,21], and it was found that PR2–5, PR8, and PR11–13 are involved in plant responses to *Fusarium* infection [2,17,22,23,24].

*Fusarium*-caused diseases are responsible for significant losses in the yield of garlic (*Allium sativum* L.)—the second most important bulbous crop in the world, with a production volume of over 30 million tons per year (http://www.fao.org/ (accessed on 5 May 2021)). During garlic cultivation and storage, *Fusarium* infection may lead to FBR of the bulbs and/or FW of the leaves [14,25]. Both diseases are caused by a specific isolate of *F. oxysporum* (f. sp. *cepae*) as well as by other *Fusarium* species, such as *F. acutatum*, *F. anthophilium*, and *F. proliferatum* [14,26]. Comparative studies of FBR-resistant and susceptible *Allium* genotypes have disclosed some of the possible biochemical and genetic mechanisms underlying plant susceptibility to FBR [27,28]. In our previous study, we have shown that garlic chitinases belonging to the PR3 family are involved in the immune response against *Fusarium* infection [28]. However, there is little information regarding the immune defense function of other PR proteins in garlic [29,30].

In the present study, we performed in silico identification and characterization of the genes belonging to the PR1, PR2, PR4, and PR5 families in the *A. sativum* genome and studied their tissue expression patterns in FBR-resistant and susceptible garlic cultivars infected with *F. proliferatum*.

## 2. Results

### 2.1. Identification of PR1, PR2, PR4, and PR5 Genes in the A. sativum Genome

**Figure 1:**
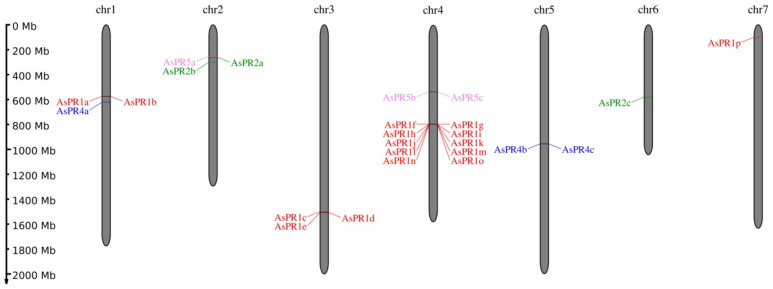
Chromosomal locations of the *AsPR1* (red), *AsPR2* (green), *AsPR4* (blue), and *AsPR5* (purple) genes. The chromosome lengths indicated on the left are based on the *A. sativum* cv. Ershuizao genome (PRJNA606385) [31]; chr, chromosome.

In silico analysis of the *A. sativum* cv. Ershuizao genome data (PRJNA606385) [31] revealed 16 genes belonging to the PR1 family, and 3 genes in each of the PR2, PR4, and PR5 families (Table 1). The *PR1* genes were identified on chromosomes 1 (*AsPR1a*, *b*), 3 (*AsPR1c-e*), 4 (*AsPR1f-o*, clustered in the 3.34 Mbp region), and 7 (*AsPR1p*). The *PR2* genes were localized on chromosomes 2 (*AsPR2a*, *b*) and 6 (*AsPR2c*), the *PR4* genes on chromosomes 1 (*AsPR4a*) and 5 (*AsPR4b*, *c*), and the *PR5* genes on chromosomes 2 (*AsPR5a*) and 4 (*AsPR5b*, *c*) (Table 1, Figure 1).

The sizes of the predicted *PR* genes varied from 438 bp (*AsPR1o*) to 1856 bp (*AsPR2c*). The *AsPR1* and *AsPR5* genes did not contain introns, whereas the *AsPR2* and *AsPR4* genes contained 1 (*AsPR4a-c*, *AsPR2a*, and *AsPR2b*) or 2 (*AsPR2c*) introns (Table 1). The coding sequences (CDSs) of the *AsPR* genes ranged from 438 bp (*AsPR1o*) to 1035 bp (*AsPR2a*), corresponding to proteins from 145 to 344 amino acid residues.

Many of the identified *PR* genes within each PR family were highly identical: *AsPR1a* and *b* (94.0%), *AsPR1c* and *e* (91.4%), *AsPR1f* and *g* (98.8%), *AsPR1h* and *m* (96.0%), *AsPR1i* and *l* (99.2%), *AsPR4a* and *c* (100%), and *AsPR5a* and *b* (98.2%).

### 2.2. Characterization of Putative PR1, PR2, PR4, and PR5 Proteins in Garlic

The predicted AsPR proteins were characterized for their physicochemical properties and functional annotation in gene ontology terms (Table 2). The molecular weight (MW) ranged from 15.5–15.55 kDa in AsPR4 proteins to 33.53–37.33 kDa in AsPR2 proteins. AsPR1 proteins significantly differed in isoelectric point (pI) (from 4.5 in AsPR1f to 9.25 in AsPR1i), whereas AsPR5 proteins demonstrated close similarity both in pI (4.71–4.74) and MW. The aliphatic index (AI), reflecting the relative number of hydrophobic residues, ranged from 55.00 in AsPR5c to 94.01 in AsPR2a, and almost all AsPR proteins were predicted to be hydrophilic (GRAVY < 0).

In terms of functional activity, the AsPR1, AsPR4, and AsPR5 proteins were associated with plant defense responses, whereas AsPR2 was associated with the carbohydrate metabolic process (Table 2). Except for AsPR2c (localized in the nucleus), all AsPR factors had an N-terminal signal peptide of 19–29 residues and were predicted to be secreted.

### 2.3. Conserved Domains in AsPR Proteins

All putative AsPR1 proteins contained a functional CAP domain (characteristic for cysteine-rich secretory proteins, antigen 5, and PR1 family members; pfam00188) (Table 2), as well as the conserved motif CxHYTQ[L/V]VWA[N/K]S[V/I]xIGC, which was also specific to the PR1 family [32]. The AsPR2 proteins contained the GH17 domain (glycosyl hydrolase family 17; pfam00332), the AsPR4 proteins contained the Barwin domain (pfam00967), and the AsPR5 proteins, the GH64-TLP-SF domain (glycoside hydrolase family 64 and thaumatin-like proteins superfamily; pfam00314) (Table 2).

### 2.4. Cis-Acting Elements in the Promoters of the AsPR1, AsPR2, AsPR4, and AsPR5 Genes

In total, 15 hormone-responsive *cis*-elements were identified: 1–12 in each *AsPR* gene (except *AsPR4a*), including elements involved in the response to abscisic acid (ABA) (*AsPR1a*, *d*, f, *i*, *k*, *l*, *n*, *AsPR2a–c*, *AsPR4b*, and *AsPR5b, c*), auxin (*AsPR1a*, *n*, *AsPR4b*, *c*, and *AsPR5b*, *c*), methyl JA (MeJA) (*AsPR1b*, *d*, *i*, *l*, *m*, *AsPR2a*, *c AsPR4b*, and *AsPR5a–c*), SA (*AsPR1b–d*, *g–i*, *l*, *m*, *o*, *p*, *AsPR2a*, *c*, *AsPR4b*, and *AsPR5a–c*), gibberellic acid (GA) (*AsPR1b*, *e*, *p*, *AsPR2c*, and *AsPR5b*, *c*), and ethylene (ET) (*AsPR1c–e*, *j–l*, *n*, and *AsPR2b*) (Table 3).

Furthermore, each *AsPR* promoter contained 2–7 of the 11 identified *cis*-elements related to abiotic stresses, such as anaerobic conditions (*AsPR1a*, *b*, *e*, *h*, *o*, *p*, *AsPR2a*, *AsPR4a*, *b*, and *AsPR5a–c*), drought (all genes except *AsPR1a*, *e–g, p*, *AsPR2a*, *AsPR4b*, and *AsPR5a*), cold (*AsPR1f*, *g*, and *AsPR5b*, *c*), heat, osmotic shock, low pH, and starvation (*AsPR1a*, *c*, *e*, *i*, *k*, *l*, *n*, *o*, *AsPR2a*, *c*, and *AsPR5a–c*), salt and heavy metals (*AsPR1d*), defense (*AsPR1g*, *i*, *p*, *AsPR2a–c*, *AsPR4a*, *c*, and *AsPR5a*), wounding, and pathogens (all genes except *AsPR1a*, *b*, *l*, *n*, *AsPR2b*, *AsPR4a*, *b*, and *AsPR5b*, *c*) (Table 3).

### 2.5. Expression Patterns of AsPR Genes

**Figure 2:**
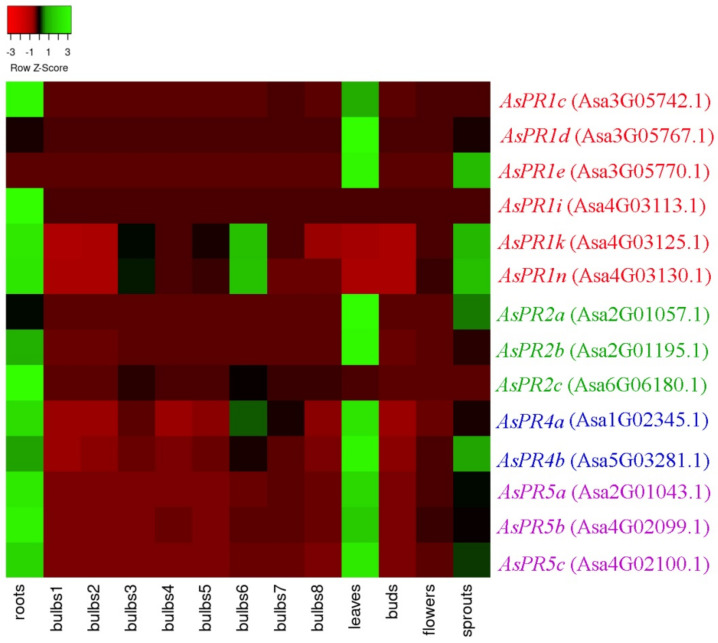
Expression heatmap of *PR* genes in *Allium sativum* cv. Ershuizao (GSE145455). The mRNA expression of *AsPR1* (red), *AsPR2* (green), *AsPR4* (blue), and *AsPR5* (purple) in the roots, bulbs (1, 2, 3, 4, 5, 6, and 7 correspond to 192-, 197-, 202-, 207-, 212-, 217-, 222-, and 227-day-old bulbs, respectively), leaves, buds, flowers, and sprouts. The color scheme indicates the gene expression gradient from low (red) to high (green).

Analysis of the *A. sativum* cv. Ershuizao transcriptome (PRJNA607255, GSE145455) [31] indicated that all of the identified *AsPR* genes were transcribed in garlic tissues, including roots, bulbs (8 developmental stages), leaves, buds, flowers, and sprouts. The maximum levels of *AsPR1* expression were detected in the roots (*AsPR1c*, *i*, *k*, *n*), leaves (*AsPR1c*–*e*), sprouts (*AsPR1i*, *k*, *n*), and stage 6 bulbs (*AsPR1k*, *n*), whereas *AsPR1k*, *n* were not expressed in stages 1 and 2 bulbs, leaves, and buds (Figure 2). A strong expression of *AsPR2* was observed in the roots (*AsPR2a–c*), leaves (*AsPR2a*, *b*), and sprouts (*AsPR2a*), of *AsPR4*, in the roots, leaves, stage 6 bulbs, and sprouts, and of *AsPR5*, in the roots, leaves, and sprouts (Figure 2). The expressions of *AsPR1a*, *b*, *h*, *l*, *m*, *o*, *p*, and *AsPR4c* were extremely low.

### 2.6. AsPR Gene Expression in Response to F. proliferatum Infection

Since the *AsPR* genes are likely to be involved in the plant defense response (Table 2), their expression was evaluated in two garlic cultivars resistant (cv. Sarmat) and susceptible (cv. Strelets) to FBR. The plants were infected with *F. proliferatum* and analyzed at two time points (24 and 96 h post-inoculation (hpi)) covering the peak of *PR* gene expression in response to hemibiotrophic pathogens [33]. The results indicate that FBR symptoms, such as white mycelium on the roots, were observed at 96 hpi only in cv. Strelets.

**Figure 3:**
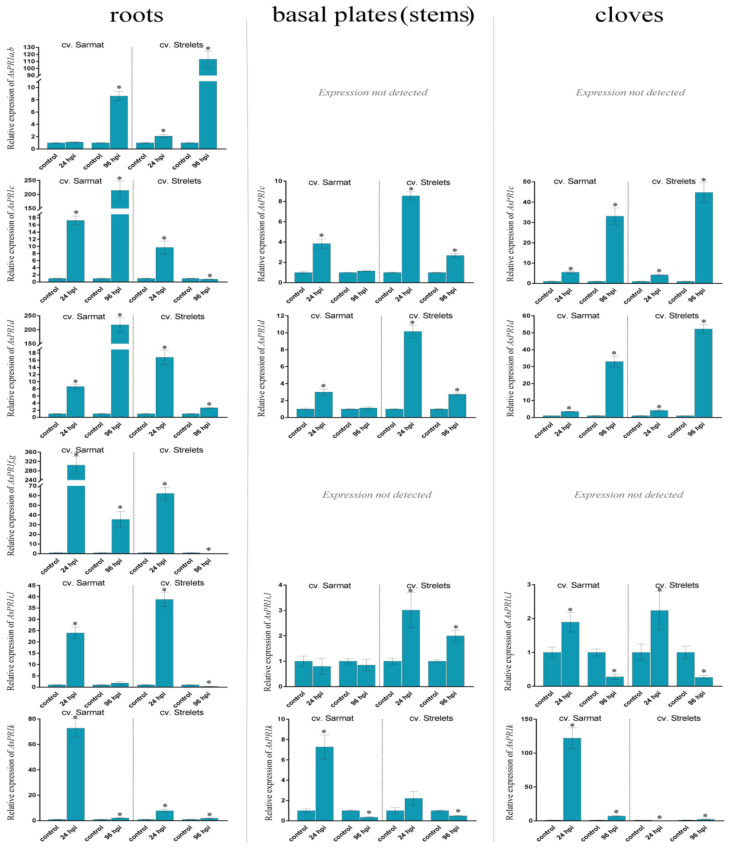
Transcription of the *AsPR1a(b)*, *AsPR1c*, *AsPR1d*, *AsPR1f(g)*, *AsPR1i(l)*, and *AsPR1k* genes in *Allium sativum* cv. Sarmat (FBR-resistant) and Strelets (FBR-susceptible) in response to *Fusarium proliferatum* infection. The plants were incubated with *F. proliferatum* conidia and analyzed for mRNA levels in the roots, stems, and cloves at 24 and 96 hpi by qRT-PCR. The data were normalized to *GAPDH* and *UBQ* mRNA levels and presented as fold change (mean ± SE) of the control taken as 1; * *p* < 0.01, compared to the uninfected control.

**Figure 4:**
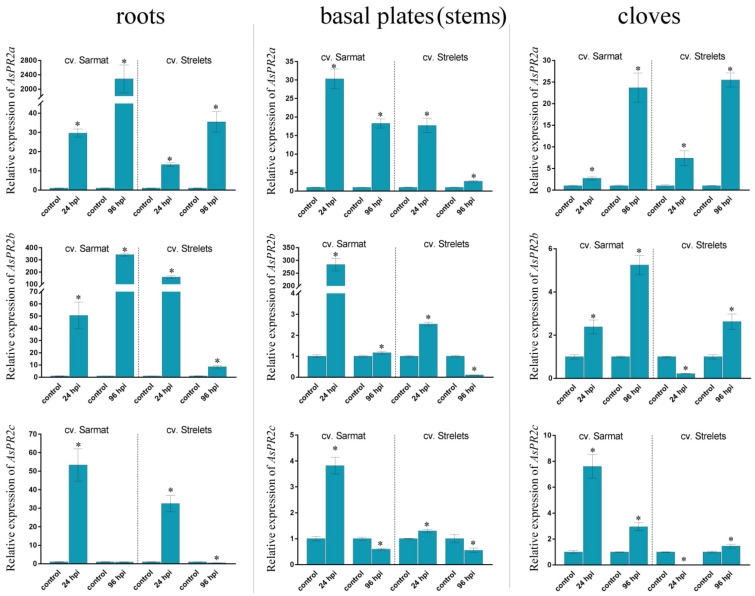
Expression of the *AsPR2a*, *b*, *c* genes in *Allium sativum* cv. Sarmat (FBR-resistant) and Strelets (FBR-susceptible) after *Fusarium proliferatum* infection. The plants were incubated with *F. proliferatum* conidia and analyzed for mRNA levels in the roots, stems, and cloves at 24 and 96 hpi by qRT-PCR. The data were normalized to *GAPDH* and *UBQ* mRNA levels and presented as fold change (mean ± SE) of the control taken as 1; * *p* < 0.01, compared to the uninfected control.

**Figure 5:**
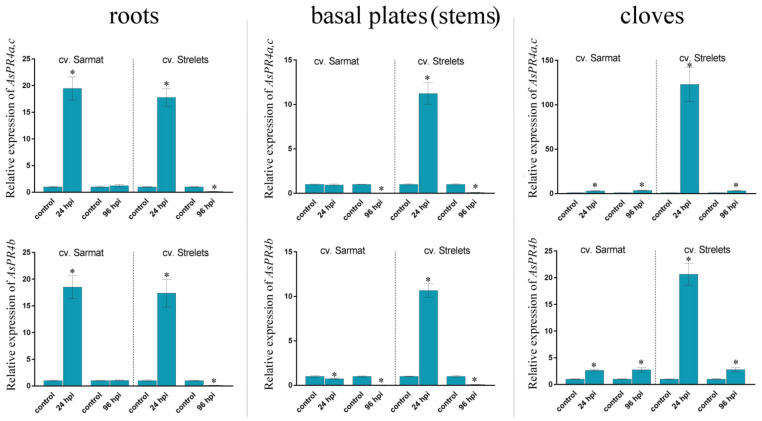
Expression of the *AsPR4a(c)* and *AsPR4b* genes in *Allium sativum* cv. Sarmat (FBR-resistant) and Strelets (FBR-susceptible) infected with *Fusarium proliferatum*. The plants were incubated with *F. proliferatum* conidia and analyzed for mRNA levels in the roots, stems, and cloves at 24 and 96 hpi by qRT-PCR. The data were normalized to *GAPDH* and *UBQ* mRNA levels and presented as fold change (mean ± SE) of the control taken as 1; * *p* < 0.01, compared to the uninfected control. Because *AsPR2a* and *AsPR4c* are 100% identical, the *AsPR4a* transcription level also includes that of *AsPR4c*.

**Figure 6:**
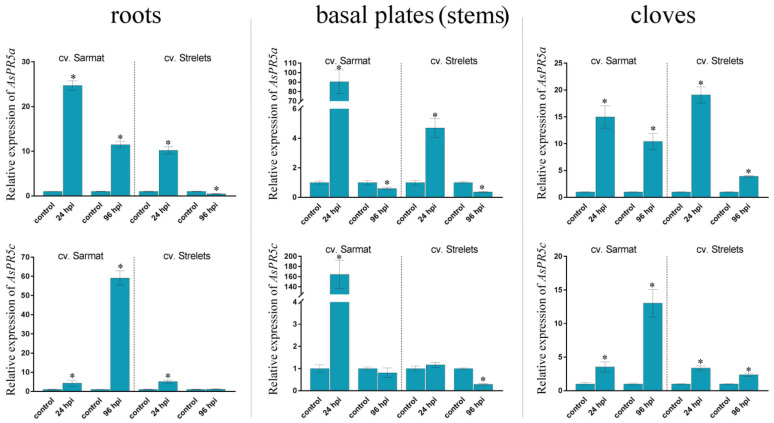
Expression of the *AsPR5a*, *c* genes in *Allium sativum* cv. Sarmat (FBR-resistant) and Strelets (FBR-susceptible) after infection with *Fusarium proliferatum*. The plants were incubated with *F. proliferatum* conidia and analyzed for mRNA levels in the roots, stems, and cloves at 24 and 96 hpi by qRT-PCR. The data were normalized to *GAPDH* and *UBQ* mRNA levels and presented as fold change (mean ± SE) of the control taken as 1; * *p* < 0.01 compared to the uninfected control.

The expression of the *AsPR* genes was compared in the roots, stems (basal plates), and cloves of the infected and control bulbs (Figure 3, Figure 4, Figure 5 and Figure 6 and Figure S1).

#### 2.6.1. AsPR1 Genes

Most of the *AsPR1* genes were expressed in the roots, stems, and cloves, except for *AsPR1a, b*, *f*, *g*, which were transcribed only in the roots, and *AsPR1p*, which was not transcribed in the analyzed organs (Figure 3).

To determine which genes of *AsPR1f* and *AsPR1g* were expressed in the tissues of the two garlic cultivars, we performed sequencing of PCR-amplified products, which revealed that only *AsPR1g* was transcribed.

Overall, the expression of *AsPR1* genes was induced by *Fusarium* infection in both cultivars, albeit to varying degrees. Thus, in cv. Sarmat, *AsPR1* genes were upregulated 8–304 times in the roots (except for *AsPR1a*, *b*), 3–7 times in the stems (except for *AsPR1i*), and 2–122 times in the cloves at 24 hpi (Figure 3). At 96 hpi, there was a 200-fold increase in the expression of *AsPR1c*, *d* and a 2–35-fold increase in that of the other *AsPR1* genes in the roots (except for *AsPR1i*, whose expression was similar to the control), whereas, in the stems, the *AsPR1* expression was significantly decreased compared to 24 hpi, mostly down to the control levels (or even lower in the case of *AsPR1k*). In the cloves, *AsPR1* transcription was increased by 33 (*AsPR1c*, *d*), or decreased by 4 (*AsPR1k*) and 17 (*AsPR1i*) times (Figure 3).

Compared to FBR-resistant cv. Sarmat, in FBR-susceptible cv. Strelets *AsPR1* genes were activated by the infection at 24 hpi to a lesser extent in the roots (except for *AsPR1i*), to a bigger extent in the stems (except for *AsPR1k*), and to a similar extent in the cloves (except for *AsPR1k*). At 24 hpi, *AsPR1* transcription was induced by 2–62 times in the roots, 2–10 times in the stems (except for *AsPR1k*), and 2–4 times in the cloves (except for *AsPR1k*, the transcription of which decreased 62 times) (Figure 3). At 96 hpi, the expression of *AsPR1a*, *b* in the roots was upregulated by 113 times, whereas that of the other *AsPR1* genes was induced or downregulated slightly; in the stems, *AsPR1* expression was increased by 2.0–2.7 times (except for *AsPR1k*, showing a 2-fold decrease), and in the cloves, it increased by 2 to 52 times (*AsPR1k*, *AsPR1c*, and *AsPR1d*) or decreased by 4 times (*AsPR1i*) (Figure 3).

#### 2.6.2. AsPR2 Genes

All three identified *AsPR2* genes were transcribed in the roots, stems, and cloves of both cultivars and were mostly upregulated in response to *F. proliferatum* infection (Figure 4). Specifically, in cv. Sarmat, the expression of the *AsPR2a*, *b*, *c* genes in all organs was significantly increased by infection (except for a decrease in *AsPR2c* expression, observed at 96 hpi in the roots and stems) (Figure 4). In cv. Strelets, all *AsPR2* genes were upregulated at 24 hpi in the roots and stems compared to the control; in the cloves, the expression of *AsPR2a* was increased, but that of *AsPR2b*, *c* decreased. At 96 hpi, the expression of *AsPR2a* was increased in all organs, whereas that of *AsPR2b* increased in the roots and cloves but decreased in the stems, and that of *AsPR2c* decreased in the roots and stems and increased in the cloves (Figure 4).

Comparison of the two cultivars revealed that, overall, the upregulation of *AsPR2* genes was much more significant in cv. Sarmat than in cv. Strelets. Thus, at 24 and 96 hpi, *AsPR2a* transcription in the roots and stems of cv. Sarmat increased by 30 and 2285, and 30 and 18 times, whereas, in those of cv. Strelets, it increased only by 13 and 35, and 18 and 2.6 times, respectively (Figure 4). *AsPR2b* transcription was increased by 50 and 343 times in the roots of cv. Strelets and by 159 and 8.5 times in those of cv. Strelets at 24 and 96 hpi, respectively. In the stems, *AsPR2b* expression was increased by 283 and 1.1 times in cv. Sarmat at 24 and 96 hpi, whereas in those of cv. Strelets, it was upregulated by 2.5 times at 24 hpi and downregulated by 10 times at 96 hpi; in the cloves, it was increased by 2.4 and 5.2 times at 24 and 96 hpi in cv. Sarmat, but decreased by 5 times at 24 hpi and increased by 2.6 times at 96 hpi in cv. Strelets (Figure 4).

The expression of *AsPR2c* gene showed a similar pattern in the roots and stems of the two cultivars. Thus, in the roots, it increased by 53 and 32 times at 24 hpi, and decreased to the control level or below at 96 hpi, whereas in the stems, it increased by 3.8- and 1.3-fold at 24 hpi, and decreased by 1.6- and 1.8-fold at 96 hpi in cv. Sarmat and cv. Strelets, respectively. However, in the cloves of cv. Sarmat, *AsPR2c* expression increased by 7.6 and 2.9 times, whereas, in those of cv. Strelets, it decreased by 83 times and increased by 1.5 times at 24 and 96 hpi, respectively (Figure 4).

#### 2.6.3. AsPR4 Genes

*AsPR4* transcription showed a similar dynamic in response to infection in the roots of both cultivars, where it was significantly upregulated at 24 hpi but dropped to or below the control levels at 96 hpi (Figure 5). In the stems of cv. Sarmat, *AsPR4* expression was either similar or significantly lower than in the control, whereas in cv. Strelets, it was markedly upregulated at 24 hpi, but dropped significantly below the control levels at 96 hpi. In the cloves, *AsPR4* transcription increased by about 3 times in cv. Sarmat and much more significantly (123-fold for *AsPR4a(c)* and 20-fold for *AsPR4b*) in cv. Strelets at 24 hpi, but not at 96 hpi (Figure 5).

These data indicate that, overall, the expression of the *AsPR4* genes followed a similar trend in the roots of both cultivars, whereas in stems and cloves, it was strongly activated at 24 hpi only in FBR-susceptible cv. Strelets, showing a stronger response to *Fusarium* infection, which is probably associated with a faster spread of pathogens compared to the FBR-resistant cv. Sarmat.

#### 2.6.4. AsPR5 Genes

*AsPR5a, c* genes were expressed in all organs of both cultivars (Figure 6), whereas *AsPR5b* was not detected.

In the roots, *AsPR5a* transcription was increased by 25- and 11-fold in cv. Sarmat, and upregulated by 10-fold but downregulated by 2-fold in cv. Strelets at 24 and 96 hpi, respectively, whereas that of *AsPR5c* was increased by 5-fold in both cultivars at 24 hpi and continued to increase at 96 hpi in cv. Sarmat (by 59-fold) but not in cv. Strelets (Figure 6). In the stems of cv. Sarmat, the levels of *AsPR5a* and *AsPR5c* mRNA were significantly increased (by 90- and 164-fold, respectively) at 24 hpi; however, at 96 hpi, they were lower or similar, respectively, to those in the uninfected control. In the stems of cv. Strelets, the expression of *AsPR5a* was upregulated by 4.7-fold and that of *AsPR5c* was similar to the control at 24 hpi, and downregulated by 2.7 (*AsPR5a*) and 3.4 (*AsPR5c*) times at 96 hpi (Figure 6). In the cloves, the expression of *AsPR5a*, *c* was upregulated at both time points in both cultivars. Thus, mRNA levels of *AsPR5a* and *AsPR5c* at 24/96 hpi were increased by 15 and 10, and 3.5 and 13 times in cv. Sarmat, and by 19 and 4 and 3.4 and 2.4 times in cv. Strelets, respectively (Figure 6).

These data indicate that overall, the expression of the *AsPR5* genes followed a trend similar to that of the other *AsPR* genes, showing stronger induction by *Fusarium* infection in resistant cv. Sarmat.

### 2.7. Cloning and Characterization of CDSs of AsPR Genes Differentially Expressed in FBR-Sensitive and Resistant Cultivars

To examine polymorphisms in *AsPR* genes between FBR-resistant (Sarmat) and FBR-sensitive (Strelets) cultivars, we amplified, cloned, and sequenced the CDSs of 9 genes (*AsPR1c*, *d*, *g*, *k*, *AsPR2a*, *b*, *c*, and *AsPR5a*, *c*) that showed the strongest transcriptional response to *F. proliferatum* infection in cv. Sarmat.

Compared to cv. Ershuizao, used as a reference, the *AsPR1g* of cv. Sarmat and Strelets did not have single nucleotide polymorphisms (SNPs), whereas the other analyzed genes contained 1–10 SNPs (Table 4). There were 13 SNPs shared by cv. Sarmat and Strelets; 5 of them were non-synonymous and led to amino acid substitutions: c. 481G > A to p. V161I in AsPR1c, c. 855G > C to p. L285F in AsPR2c, c. 403G > A to p. G135S and c. 620T > C to p. I207T in AsPR5a, and c. 647A > T to p. D216V in AsPR5c (Table 4). Cultivar-specific SNPs were identified in *AsPR1d* (c. 200T > G leading to p. I67R) of cv. Sarmat and in *AsPR1c* (c. 379G > C to p. V127L), *AsPR2a* (c. 35T > C to p. L12S and c. 559A > C to p. I187L), and *AsPR5a* (c. 142T > A to p. S48T) in cv. Strelets (Table 4). According to PROVEAN, p. V127L and p. G135S were predicted to be potentially deleterious substitutions, whereas the rest were neutral.

## 3. Discussion

Fungi dominate among plant parasites in terms of the number of diseases they cause [34]. Infection with the most viable and destructive fungi of the *Fusarium* genus is accompanied by wilting and the appearance of mycelium and brown necrotic areas (accumulation of dead plant cells); at the molecular level, it is characterized by the production of camalexin and ROS, deposition of callose, and changes in the activity of PR proteins [29,34]. In *A. sativum*, infection by *Fusarium* spp. occurs through the roots and basal plates (stems), causing FBR and FW [26,35,36], which are responsible for 60% of the global garlic crop losses at pre- and post-harvest stages [29].

Genes of the PR1, PR2, PR3, PR4, PR5, PR12, and PR13 families encode proteins with antifungal activity, in particular, against *Fusarium* spp. [2]. In *A. sativum*, the expression of *PR1*, *PR3*, and *PR5* genes is considered a positive marker of plant resistance to *F. oxysporum* f. sp. *cepae* [29]. Furthermore, *PR3* genes *AsCHI2*, *AsCHI3*, *AsCHI5*, and *AsCHI7,* encoding GH19 class I chitinases, have been shown to be involved in the response to *F. proliferatum* attack [28].

In this study, we identified and characterized 25 *PR* genes in *A. sativum* cv. Ershuizao: 16 *PR1*, 3 *PR2*, 3 *PR4*, and 3 *PR5*, which encode CAP-domain proteins, β-1,3-glucanases, Barwin-domain proteins, and thaumatin-like proteins, respectively (Table 1). CDSs of 4 *AsPR1*, 3 *AsPR2*, and 2 *AsPR5* genes were amplified in FBR-resistant cv. Sarmat and FBR-susceptible cv. Strelets; these genes are orthologs of *A. sativum PR1* (JN011451.1) and *PR5* (KP782043.1), and *A. thaliana PR2* (NM_115587.2) and *PR4* (NM_111344.6), respectively. The presence of multiple paralogs in each PR family suggests functional redundancy or divergence among PR factors. Paralogs are mostly tandemly clustered on chromosomes (Figure 1), suggesting that *AsPR* gene families originated as a result of evolutionary tandem duplications.

Our results indicate that the promoters of the *AsPR* genes contain 15 *cis*-regulatory elements involved in the response to hormones such as ABA, SA, MeJA, auxin, ET, and GA, as well as 12 elements associated with immune defense and response to elicitors and stresses such as anaerobic conditions, dehydration, low and high temperature, salinization, heavy metals, and wounding (Table 3). This regulatory profile is similar to those of class I chitinase genes of *A. sativum* (*AsPR3*) [28] and *B. rapa* [37]. Stress-responsive elements STRE, W-box, and WUN-motif, known to mediate pathogen- and/or elicitor-inducible transcription of chitinase genes [33,38,39], were found in the promoters of *AsPR1*, *AsPR2*, and *AsPR5* (STRE), *AsPR1f*, *g* (W-box), and *AsPR1*, *AsPR2*, and *AsPR4* (WUN-motif), confirming that the four identified PR families should be involved in the response to pathogens, including fungi.

*Fusarium* fungi are hemibiotrophs and pass through a biotrophic phase before switching to necrotrophy [34]. As such, they first elicit systemic (SAR) and then local (LAR) defense responses associated with antagonistic SA and JA signaling, respectively, which activates the transcription of *PR1*, *PR2*, and *PR5* (SA), and *PR3*, *PR4*, and *PR11* (JA) [2,40]. Because *AsPR* genes contain SA-responsive (*AsPR1b–d*, *g–i*, *l*, *m*, *o*, *p*, *AsPR2a*, *c*, *AsPR4b*, and *AsPR5a–c*) and JA-responsive (*AsPR1b*, *d*, *i*, *l*, *m*, *AsPR2a*, *c*, *AsPR4b*, and *AsPR5a–c*) elements in their promoters (Table 3), they could be involved in both the SAR and the LAR of garlic to *Fusarium* attack: *ASPR1*, *AsPR2*, and *AsPR5* genes at the biotrophic phase, and *AsPR4* genes at the necrotrophic phase. The presence of ET-responsive elements in *AsPR1c–e*, *j–l*, *n* and *AsPR2b*, and ABA-responsive elements in *AsPR1a*, *d*, *f*, *i*, *k*, *l*, *n*, *AsPR2a–c*, *AsPR4b*, and *AsPR5b*, *c* (Table 3) further confirms the potential roles of these genes in LAR, since ET and ABA synthesis is induced in response to necrotrophic pathogens [40,41]. Moreover, the presence of GA-responsive elements in *AsPR1b*, *e*, *p*, *AsPR2c*, and *AsPR5b*, *c* and auxin-responsive elements in *AsPR1a*, *n*, *AsPR4b*, *c*, and *AsPR5b*, *c* (Table 3) supports the involvement of these genes in LAR because the crosstalk of auxins and GAs with JA mediates the defense against pathogens [42].

Our results indicate that *Fusarium* infection activates *AsPR* transcription primarily in the roots (Figure 3, Figure 4, Figure 5 and Figure 6) representing the first barrier to the penetration of soil-dwelling fungi [43], which is consistent with the role of PR proteins in the antifungal defense. However, there was no clear correlation between the timing of gene activation in response to infection (24 or 96 hpi) (Figure 3, Figure 4, Figure 5 and Figure 6) and their putative role in SAR or LAR, especially since the infection signs at 96 hpi were limited to the appearance of mycelium, suggesting that *Fusarium* infection was still at its biotrophic phase and did not yet proceed to the necrotrophic phase.

At the same time, we found significant variations in gene expression between FBR-resistant and susceptible cultivars (Figure 3, Figure 4, Figure 5 and Figure 6). Compared to the susceptible cv. Strelets, in the resistant cv. Sarmat, the *AsPR5* and *AsPR2* genes showed stronger activation (the latter especially in the roots and stems), whereas *AsPR4* showed weaker activation in the stems and cloves at 24 hpi (Figure 4, Figure 5 and Figure 6). The regulatory mode of the multiple identified *AsPR1* genes differed depending on the organ; *AsPR1c*, *d, g*, *k* showed stronger activation in the roots (the latter also in the stems and cloves), whereas *AsPR1a*, *b, i* had weaker activation in the roots and *AsPR1d, i*—in the stems of cv. Sarmat (Figure 3). Given that the encoded PR proteins provide plant protection against fungal infection, these data suggest the mRNA expression profiles of *AsPR1c*, *d*, *g*, *k*, *AsPR2b* and *AsPR5a*, *c* (in roots), as well as *AsPR4a(c)*, *b*, and *AsPR2c* (in stems and cloves), and may serve as a marker of resistance to FBR in garlic cultivars. Thus, sterol-binding PR1 proteins suppress the proliferation of fungal cells because of sterol extraction from their membranes [44], PR2 β-1,3-glucanases, in combination with chitinases, hydrolyze fungal cell walls [45], PR4 Barwin-domain proteins directly inhibit hyphal growth [46], and PR5 thaumatin-like proteins may increase membrane permeability through the destruction of β-1,3-glucans or the inhibition of fungal enzymes, such as xylanases [47]. Compared to other *AsPR* genes, the *AsPR1* family appears to have undergone a particularly high level of expansion (Figure 1). Considering the activation of the expression of at least half of the *AsPR1* genes in response to *Fusarium* infection (Figure 3), the participation of CAP-domain proteins in immune defense may be critical. The presence of a large number of active AsPR1 paralogs suggests their redundancy in the immune response, which may insure the plant against fungal infection even in the event of knockout of individual family members.

The *AsPR1e*, *h*, *j*, *l*–*p*, and *AsPR5b* genes were not functional in cv. Sarmat and Strelets, since their mRNA was not detected irrespectively of *F. proliferatum* infection; however, *AsPR1n* and *AsPR5b* transcripts were observed in the roots and bulbs of cv. Ershuizao (Figure 2), suggesting that these genes are expressed in a cultivar-specific manner.

## 4. Materials and Methods

### 4.1. In Silico Identification and Structural Characterization of PR1, PR2, PR4, and PR5 Genes in the Allium Sativum Genome

The search of *PR1*, *PR2*, *PR4*, and *PR5* genes was performed in the *A. sativum* cv. Ershuizao transcriptome (NCBI accession number: PRJNA607255) and whole-genome shotgun contigs (PRJNA606385) [31]. Sequences of garlic *AsPR1* (JN011451.1) and *AsPR5* (KP782043.1) genes and *A. thaliana PR2* (AT3G57270; NM_115587.2) and *PR4* (AT3G04720; NM_111344.6) genes were used as references. The selected sequences contained start and stop codons and full-length catalytic domains. Multiple sequence alignment and structural analysis of *AsPR* genes and the encoded proteins were conducted with MEGA 7.0.26 [48]. To predict exon–intron structures, *AsPR* genes and CDSs were analyzed with GSDS v2.0 [49]. The predicted proteins were characterized by MW, pI, AI, and GRAVY (ExPASy ProtParam; https://web.expasy.org/protparam/; accessed on 1 March 2021), conserved domains, sites, and motifs (NCBI-CDD, https://www.ncbi.nlm.nih.gov/cdd; accessed on 1 March 2021), biological processes (PANNZER2; http://ekhidna2.biocenter.helsinki.fi/sanspanz/; accessed on 1 March 2021), subcellular localization (BaCello; http://gpcr2.biocomp.unibo.it/; accessed on 1 March 2021), the functional importance of residue substitutions (PROVEAN; [50]), and signal peptide cleavage sites (SignalP 5.0; http://www.cbs.dtu.dk/services/SignalP/; accessed on 1 March 2021). The chromosomal localization map was drawn using MG2C v. 2.1 (http://mg2c.iask.in/mg2c_v2.1/; accessed on 1 March 2021).

### 4.2. In Silico mRNA Expression Analysis

The expression of *AsPR* genes in *A. sativum* cv. Ershuizao tissues (roots, bulbs, stems (basal plates), leaves, buds, flowers, and sprouts) was determined based on RNA-seq data (ID: PRJNA607255), normalized using Fragments Per Kilobase of transcript per Million mapped reads (FPKM) assay [31], and visualized using Heatmapper [51]; only transcripts with an average FPKM value of ≥10 in at least one of the organs were used for heatmap construction.

### 4.3. Fungi, Plant Material, and F. proliferatum Infection Assay

*F. proliferatum* was kindly provided by the Group of Experimental Mycology, Winogradsky Institute of Microbiology (Research Center of Biotechnology of the RAS, Moscow, Russia). The strain was previously isolated from cv. Strelets bulbs; the pathogenicity test showed that the first signs of the disease appeared on the surface of the treated cloves after 5 days of infection [52].

Accessions of *A. sativum* cv. Sarmat and cv. Strelets, resistant and susceptible to FBR, respectively, were kindly provided by the Federal Scientific Vegetable Center (Moscow region, Russia). The choice of cultivars was based on the similarity of morphological characteristics in order to exclude their influence on experimental results [28].

The number of cloves (6) used per biological replicate in the *Fusarium* infection assay was based on that of cloves in a bulb (5–7 for cv. Strelets and 7–11 for cv. Sarmat). In total, 6 bulbs of each cultivar were used, and 6 cloves from each bulb were processed: 3 for the experiment and 3 for control.

A total of 12 cloves of each cultivar were sterilized in 70% ethanol for 3 min, rinsed with sterile water, placed in Petri dishes with wet filter paper, and incubated at +25 °C in the dark. After 36 h, active root growth was observed and half of the cloves were infected by soaking in *F. proliferatum* conidial suspension (~10^6^ conidia ml^−1^) for 5 min, as previously described [35]). The infected cloves (*n* = 6) were transferred to fresh Petri dishes and incubated at +25 °C in the dark; uninfected cloves (*n* = 6) were used as a control. The experiment was performed in 3 biological and 2 technical replicates for the infected and uninfected plants per each time point. The roots, stems, and cloves were collected at 24 hpi and 96 hpi, frozen in liquid nitrogen, and stored at −80 °C until analysis. The time points (24 and 96 hpi) were chosen based on the reported peak of *PR* gene expression, which was observed 1–3 days after inoculation with hemibiotrophic pathogens [53].

### 4.4. RNA Extraction and Quantitative Real-Time PCR (qRT-PCR) Analysis

Total RNA was extracted from individual roots, stems, and cloves (0.5 g of each tissue) using the RNeasy Plant Mini Kit (QIAGEN, Hilden, Germany), purified from genomic DNA (RNase free DNase set; QIAGEN, Hilden, Germany), qualified by gel electrophoresis, and used for first-strand cDNA synthesis (GoScript Reverse Transcription System; Promega, Madison, USA) with an oligo-dT primer. The RNA and cDNA concentrations were quantified by fluorimetry (Qubit^®^ Fluorometer, Thermo Fisher Scientific, Waltham, MA, USA) and qRT-PCR was performed in a CFX96 Real-Time PCR Detection System (Bio-Rad Laboratories, Hercules, USA) with 3.0 ng of cDNA, SYBR Green RT-PCR mixture (Syntol, Moscow, Russia), and specific primers (Table 5). Gene-specific primers were designed to amplify partial coding sequences; the primers were selected for the most variable parts of the CDS. Given the high similarity (over 98%) of the coding sequences of some paralogous genes (*AsPR1a/b*; *AsPR1f/g*; *AsPR1i/l*; *AsPR4a/c*; *AsPR5a/b*), primers that amplify both genes were selected for each pair. The following cycling conditions were used: initial denaturation at 95 °C for 5 min, 40 cycles of denaturation at 95 °C for 15 s, and annealing/extension at 60 °C for 40 s.

*AsPR* gene expression was normalized using two reference garlic genes, *GAPDH* [54] and *UBQ* [55], and the qRT-PCR results were statistically analyzed with Graph Pad Prism version 8 (GraphPad Software Inc., San Diego, CA, USA; https://www.graphpad.com/scientific-software/prism/ (accessed on 26 April 2021)). The unpaired *t*-test was applied to assess differences in gene expression; *p* < 0.01 was considered to indicate statistical significance. The data were expressed as the mean ± standard error (SE) based on 3 technical replicates of 3 biological replicates for each combination of cDNA and primer pairs.

### 4.5. Gene Identification

To amplify the CDSs of *AsPR* genes from garlic cultivars, gene-specific primers were designed based on *A. sativum* cv. Ershuizao transcriptomic data (NCBI project accession number: PRJNA607255) (Table 5). A manual revision of sequence polymorphisms and an additional evaluation were performed using Primer3 (http://frodo.wi.mit.edu/primer3/; accessed on 15 March 2021). cDNA from the roots of a single plant of each cultivar was used as a template (30 ng) for PCR amplification with the following conditions: initial denaturation at 95 °C for 5 min and 35 cycles of denaturation at 95 °C for 30 s, primer annealing at 55 °C for 30 s, and extension at 72 °C for 2 min followed with a final extension at 72 °C for 5 min. The amplified PCR products of the expected size were purified by using the QIAEX^®^ II Gel Extraction kit (QIAGEN, Hilden, Germany), cloned in the pGEM^®^-T Easy vector (Promega, Madison, WI, USA), and sequenced (3–5 clones for each accession) on an ABI Prism 3730 DNA Sequencer (Applied Biosystems, Waltham, MA, USA) using the designed primers (Table 5).

### 4.6. Promoter and 5′-UTR Analysis

The search of specific *cis*-elements in promoters and 5′-UTRs (1.0 kb regions upstream of the initiation codon) was performed using the PlantCARE database, which provides an evaluation of *cis*-regulatory elements, enhancers, and repressors [56]; (http://bioinformatics.psb.ugent.be/webtools/plantcare/html/; accessed on 10 March 2021).

## 5. Conclusions

We identified and characterized 25 genes of the PR1, PR2, PR4, and PR5 families in *A. sativum* cv. Ershuizao and cloned 4 *AsPR1*, 3 *AsPR2*, and 2 *AsPR5* homologs from garlic cultivars resistant and susceptible to FBR. The *AsPR* gene promoters contained hormone- and stress-responsive elements, including those associated with the response to fungal pathogens and their elicitors, indicating that the putative garlic PR proteins AsPR1a, c, e, f, j, k, n, AsPR2b, and AsPR4c may participate in LAR, whereas AsPR1b–d, i, l, p, AsPR2a, c, AsPR4b, and AsPR5a–c participate in both LAR and SAR. The comparison of *AsPR* transcriptional profiles in FBR-resistant and susceptible garlic cultivars infected with *F. proliferatum* suggests that the expression profile of *AsPR5a*, *c*, *AsPR2b*, and *AsPR1c*, *d*, *g*, *k* (in roots), as well as *AsPR4a(c)*, *b* and *AsPR2c* (in stems and cloves), could define the difference in the PR-mediated response to *Fusarium* infection between resistant and susceptible plants. Our results provide useful insights into the functions of *PR* genes in *A. sativum* and *Allium* plants in general, and may be used in breeding programs to increase the resistance of *Allium* crops to *Fusarium* infections.

## Figures and Tables

**Table 1:** *PR1*, *PR2*, *PR4,* and *PR5* genes identified in the genome of *A. sativum* cv. Ershuizao.

Gene	Localization	Length (bp)	Number of Exons	CDS (bp)	Protein (aa)	Transcript ID [31]
PR1 family						
*AsPR1a*	ch1:597808382-597808867 (+)	486	1	486	161	Asa1G02133.1
*AsPR1b*	ch1:597852310-597852792 (+)	483	1	483	160	Asa1G02134.1
*AsPR1c*	ch3:1569741035-1569741535(−)	501	1	501	166	Asa3G05742.1
*AsPR1d*	ch3:1574050049-1574050546 (+)	498	1	498	165	Asa3G05767.1
*AsPR1e*	ch3:1575214795-1575215280 (−)	486	1	486	161	Asa3G05770.1
*AsPR1f*	ch4:828639577-828640059 (−)	483	1	483	160	Not detected
*AsPR1g*	ch4:828659513-828659995 (−)	483	1	483	160	Not detected
*AsPR1h*	ch4:829079571-829080095 (−)	525	1	525	174	Asa4G03112.1
*AsPR1i*	ch4:829255226-829255750 (−)	525	1	525	174	Asa4G03113.1
*AsPR1j*	ch4:829291631-829292125 (−)	495	1	495	164	not detected
*AsPR1k*	ch4:831436248-831436742 (−)	495	1	495	164	Asa4G03125.1
*AsPR1l*	ch4:831555457-831555981 (+)	525	1	525	174	Asa4G03126.1
*AsPR1m*	ch4:831614218-831614742 (+)	525	1	525	174	Asa4G03127.1
*AsPR1n*	ch4:831977068-831977562 (+)	495	1	495	164	Asa4G03130.1
*AsPR1o*	ch4:831982650-831983087 (+)	438	1	438	145	Asa4G03131.1
*AsPR1p*	ch7:103854549-103855043(−)	495	1	495	164	Asa7G00352.1
PR2 family						
*AsPR2a*	ch2:272017011-272018179 (−)	1169	2	1035	344	Asa2G01057.1
*AsPR2b*	ch2:311576151-311577239 (−)	1089	2	990	329	Asa2G01195.1
*AsPR2c*	ch6:605851704-605853559 (−)	1856	3	945	314	Asa6G06180.1
PR4 family						
*AsPR4a* ^1^	ch1:652401564-652402126 (+)	563	2	444	147	Asa1G02345.1
*AsPR4b*	ch5:998122740-998123259 (+)	520	2	444	147	Asa5G03281.1
*AsPR4c* ^1^	ch5:999259985-999260547 (+)	563	2	444	147	Asa1G02345.1
PR5 family						
*AsPR5a*	ch2:266379091-266379753 (+)	663	1	663	220	Asa2G01043.1
*AsPR5b*	ch4:561214533-561215195 (+)	663	1	663	220	Asa4G02099.1
*AsPR5c*	ch4:561229259-561229921 (+)	663	1	663	220	Asa4G02100.1

^1^ These two sequences are 100% identical.

**Table 2:** Characteristics of PR1, PR2, PR4, and PR5 proteins predicted in *A. sativum* cv. Ershuizao.

Protein Symbol	MW (kDa)	pI	AI	GRAVY	Signal Peptide	Catalytic Domain	Subcellular Localization	Biological Process
PR1 family								
AsPR1a	17.34	7.55	76.40	0.038	1–23	CAP (31–149)	Secretory	Defense response (GO:0006952), response to biotic stimulus (GO:0009607)
AsPR1b	17.15	6.78	77.50	0.065	1–22	CAP (30–148)		
AsPR1c	17.84	5.99	72.77	−0.135	1–27	CAP (35–154)		
AsPR1d	17.60	6.14	75.64	−0.155	1–26	CAP (34–153)		
AsPR1e	17.14	6.48	68.94	−0.329	1–26	CAP (34–149)		
AsPR1f	17.51	4.50	63.44	−0.319	1–19	CAP (27–145)		
AsPR1g	17.45	4.63	63.44	−0.300				
AsPR1h	19.38	5.33	61.15	−0.533	1–23	CAP (31–149)		
AsPR1i	19.62	9.25	71.72	−0.487				
AsPR1j	18.10	7.58	61.34	−0.412	1–19	CAP (27–145)		
AsPR1k	18.04	8.20	61.34	−0.393				
AsPR1l	19.58	9.21	71.72	−0.485	1–23	CAP (31–149)		
AsPR1m	19.35	6.41	64.54	−0.501				
AsPR1n	18.07	8.20	59.57	−0.407	1–19	CAP (27–145)		
AsPR1o	16.05	7.59	68.69	−0.246				
AsPR1p	18.24	6.27	85.00	−0.271	1–25	CAP (34–152)		
PR2 family								
AsPR2a	34.97	7.67	94.01	0.045	1–21	GH17 (22–329)	Secretory	Carbohydrate metabolic process (GO:0005975)
AsPR2b	37.33	5.14	92.44	−0.021	1–29	GH17 (30–343)	Secretory	
AsPR2c	33.53	6.41	91.02	−0.008	n/d	GH17 (5–312)	Nucleus	
PR4 family								
AsPR4a	15.55	5.54	73.74	−0.078	1–25	Barwin (31–145)	Secretory	Defense response to fungus (GO:0050832), defense response to bacterium (GO:0042742)
AsPR4b	15.5	6.22	79.05	−0.029				
AsPR4c	15.55	5.54	73.74	−0.078				
PR5 family								
AsPR5a	23.46	4.71	58.09	−0.085	1–21	GH64-TLP-SF (28–220)	Secretory	Defense response (GO:0006952)
AsPR5b	23.50	4.71	55.86	−0.126				
AsPR5c	23.59	4.74	55.00	−0.164				

MW, molecular weight; pI, isoelectric point; AI, aliphatic index; GRAVY, grand average hydropathy; n/d, not detected.

**Table 3:** Regulatory *cis*-elements found in silico in the promoters of the *PR1*, *PR2*, *PR4*, and *PR*5 genes from *Allium sativum* cv. Ershuizao.

Motif	Response to ^1^	Number of Elements in Promoters																								
		*AsPR1a*	*AsPR1b*	*AsPR1c*	*AsPR1d*	*AsPR1e*	*AsPR1f*	*AsPR1g*	*AsPR1h*	*AsPR1i*	*AsPR1j*	*AsPR1k*	*AsPR1l*	*AsPR1m*	*AsPR1n*	*AsPR1o*	*AsPR1p*	*AsPR2a*	*AsPR2b*	*AsPR2c*	*AsPR4a*	*AsPR4b*	*AsPR4c*	*AsPR5a*	*AsPR5b*	*AsPR5c*
**Hormone Response**																										
ABRE	ABA	1			3		1			2		3	2		2			4	1	3		2			2	2
ABRE3a					1		1			1			1					1	1	1					1	1
ABRE4					1		1			1			1					1	1	1					1	1
CARE																						1				
AUXRR-core	Auxin																								1	1
AuxRE																									1	1
TGA-element		1													1							1	1			
CGTCA-motif	MeJA		1		1					1			1	1				2		2		2		1	1	1
TGACG-motif			1		1					1			1	1				1		2		2		1	1	1
AS-1	SA		1		1					1			1	1				1		2		2		1	1	1
TCA-element									2							1	2									
P-box			2	1				1														1				
TATC-box	GA					1																				
GARE-motif			1														1			1					1	1
ERE	ET			1	1	1					1	1	1		1				2							
**Stress Response**																										
ARE	Anaerobic conditions	1	1			1			1							1	1	2			1	2		1	2	2
DRE1/DRE core	Drought		1																							
MBS			1	1	1				2	2	3	2	2	1	2	3			3	1	1		1		1	1
LTR	Cold						1	1																	1	1
STRE	Heat, osmotic shock, low pH, starvation	4		1		1				1		1	1		1	1		2		1				1	1	1
F-box	Salt, heavy metals				1																					
TC-rich repeats	Defense							1		1							1	2	1	1	1		1	1		
W-box	Wounding, pathogens						1	2																		
Wun-motif									1	1	1	1		2		1	1	1		1			1			
WRE3				1	1	1		1																1		
box S									1					1												

^1^ ABA, abscisic acid; MeJA, methyl jasmonate; SA, salicylic acid; GA, gibberellic acid; ET, ethylene.

**Table 4:** Polymorphisms in the *AsPR* CDS in cv. Sarmat and Strelets compared to cv. Ershuizao.

Gene	cv. Sarmat	cv. Strelets
*AsPR1c*	c. 481G > A (p. V161I)	c. 379G > C (p. V127L), c. 417T > C, c. 481G > A (p. V161I)
*AsPR1d*	c. 117A > G, c. 200T > G (p. I67R)	
*AsPR1k*		c. 462A > G
*AsPR2a*	c. 291T > C	c. 35T > C (p. L12S), c. 559A > C p. I187L)
*AsPR2b*	c. 57A > G	
*AsPR2c*	c. 219C > A; c. 855G > C (p. L285F)	
*AsPR5a*	c. 126T > C, c. 159G > C, c. 279A > G, c. 285C > T, c. 403G > A (p. G135S), c. 468G > C, c. 620T > C (p. I207T)	
*AsPR5c*	c. 516T > C, c. 647A > T (p. D216V)	

**Table 5:** The list of primers used in the study.

Gene	Primer Sequence (5′→3′)	Application
*AsPR1a, b*	ATGGAACACGACACTGGCAG GCATACTGACCAGAGTAACTGG	Gene expression analysis (qRT-PCR)
*AsPR1c*	GGCGGTCCTTATGGTGAAA GCCAGGGTCACATGTGTTA	
*AsPR1d*	GGCGGTCCTTATGGTGAAA CCAGGGTCACATGTGTTGCT	
*AsPR1f, g*	CGATCACCACCGCAGTTCA GCGTAGTTCTGTGCGTAATCAG	
*AsPR1i*	TATGGGGAGAACCTATTCGC AATCTTRACCGACTTAGCCCA	
*AsPR1k*	GTGTCCGAGAAGCGGTACTAT AGCCGCCAGTGTTGCACC	
*AsPR1p*	GTCGCAAAATACGCGCAAAGTT GTACTTCACGACATCGGCATC	
*AsPR2a*	GCTAGAAACCATATCGTTGCCT GCATACCGTAGCATACTCCGA	
*AsPR2b*	GGTCGCATTTCTCCTAGGCAT GCGTCGCCTGCTGATGGAA	
*AsPR2c*	GGCCCATTGTCCAGTTCTTG AGGCGCCGTGAATAATGCGTA	
*AsPR4a*	ATGCCGGCATGTCCCTCG GTCTATGATCCTCACCGTCGTT	
*AsPR4b*	ATGCCGGCATGTCCCTCG GGTCTATGATCCTCACCGTCAA	
*AsPR5a*	CATCCGGACACGGCAGCT TCCATGTACTGCTTCAGAGCG	
*AsPR5c*	GCAAGCAGCTCAACTCAGGA GCCGGTCTGACATCTTCCA	
*AsPR1c*	ATGGGATCAATCAGTAGTTATA AACGACTGAGTACTCTCAGT	Gene amplification
*AsPR1d*	ATGGGATCGACCAGTACTTG TAACGTCGTAGTTGTAACGAC	
*AsPR1f, g*	ATGAAAACGTCATTTCTCTTC ATAAATAGCAGTACACACATAA	
*AsPR1k*	GCTCAAATTACAATGAAAACGTT CTGTCTGTTTCAGCATGCA	
*AsPR2a*	TGTGCACCATCGAATTACCTTC CTCTGTCTCCCTTAATAGTAC	
*AsPR2b*	AAATGCAAGCAAGGAAGCTTG CCCTGGTACATTCATAGTTAAC	
*AsPR2c*	TTGAAATGGTCATGCATGCCT AAACAGGGCACACATGCAAG	
*AsPR5a*	ATGTCGACCCAAATTACAGTC ACTCAATCCAAGAAYACAGTTC	
*AsPR5c*	ATGTCGACCCAAATTACAGTC CTGCAAACTAATTATTCGGTGA	

## Data Availability

PR genes coding sequences of *A. sativum* cv. Sarmat/cv. Strelets were deposited in NCBI: *AsPR1c* (MZ216000/MZ216001), *AsPR1d* (MZ216002/MZ216003), *AsPR1g* (MZ216004/MZ216005), *AsPR1k* (MZ216006/MZ216007), *AsPR2a* (MZ216008/MZ216009), *AsPR2b* (MZ216010/MZ216011), *AsPR2c* (MZ216012/MZ216013), *AsPR5a* (MZ216014/MZ216015), *AsPR5c* (MZ216016/MZ216017).

## Supplementary Materials

The following are available online at https://www.mdpi.com/article/10.3390/ijms22136688/s1: Figure S1: Expression of *AsPR1* (**a**), *AsPR2* (**b**), *AsPR4* (**c**) and *AsPR5* (**d**) mRNA in *Allium sativum* cv. Sarmat (FBR-resistant) and Strelets (FBR-susceptible) infected with *Fusarium proliferatum.* The plants were incubated with *F. proliferatum* conidia and analyzed for mRNA levels in the roots, stems (basal plate), and cloves at 24 and 96 hpi by qRT-PCR. The data were normalized to *GAPDH* and *UBQ* mRNA levels and presented as the mean ± SE (*n* = 3). * *p* < 0.01 compared to the same tissue in the other cultivar: blue asterisk – gene expression level in cv. Sarmat is higher than that in cv. Strelets; red asterisk – gene expression level in cv. Strelets is higher than that in cv. Sarmat.
