# Supplementary material for: Pathogenesis-Related Genes of PR1, PR2, PR4, and PR5 Families Are Involved in the Response to Fusarium Infection in Garlic (Allium sativum L.)
Source: Int J Mol Sci. 2021 Jun 22;22(13):6688. doi: 10.3390/ijms22136688 (PMC8268425; doi:10.3390/ijms22136688)
Supplement: Supplementary file 1 [file ijms-22-06688-s001.zip › ijms-1245333-supplementary.pdf]

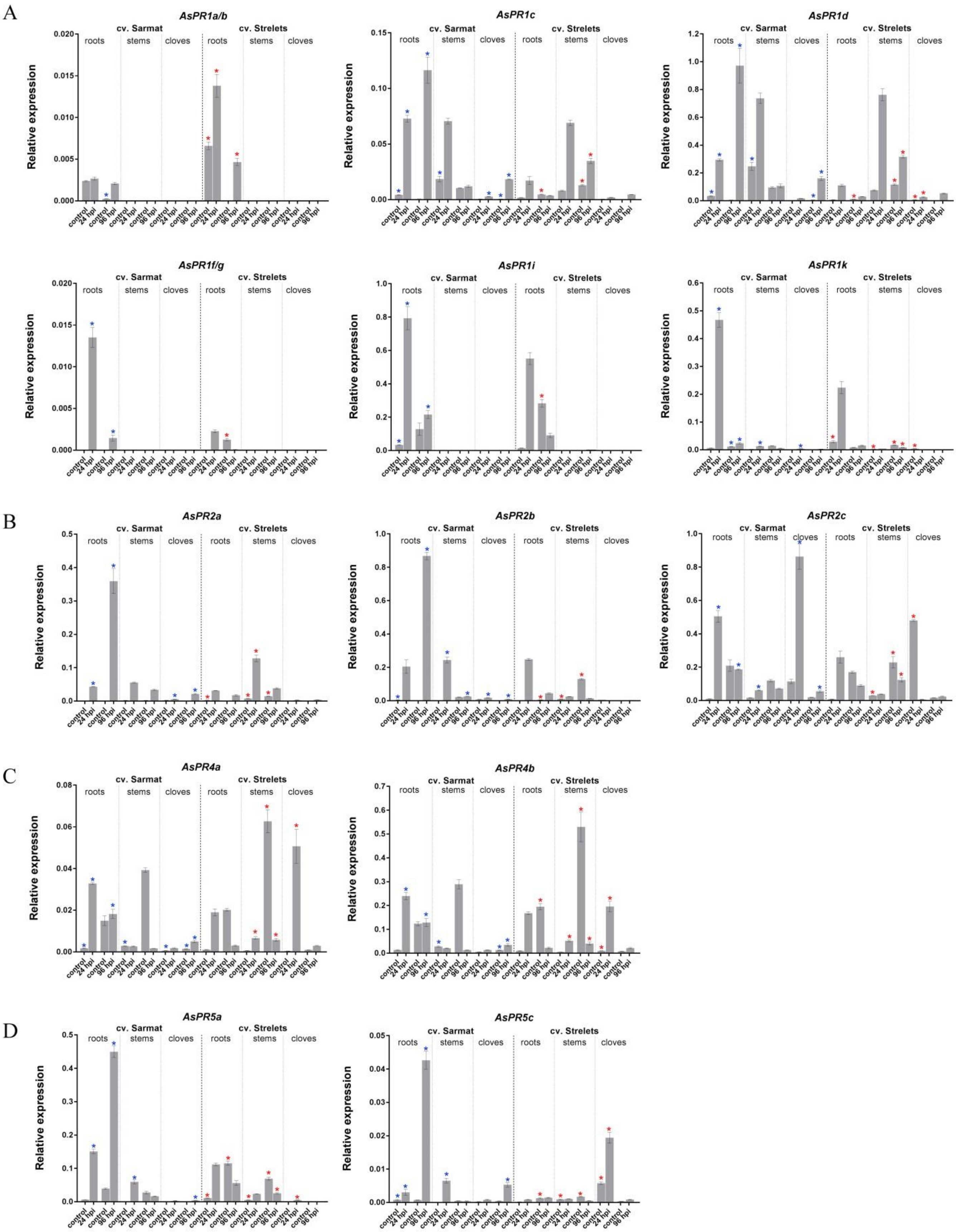


**Figure S1.** Expression of *AsPR1* (**A**), *AsPR2* (**B**), *AsPR4* (**C**) and *AsPR5* (**D**) mRNA in *Allium sativum* cv. Sarmat (FBR-resistant) and Strelets (FBR-susceptible) infected with *Fusarium proliferatum*. The plants were incubated with *F. proliferatum* conidia and analyzed for mRNA levels in the roots, stems (basal plate), and cloves at 24 and 96 hpi by qRT-PCR. The data were normalized to *GAPDH* and *UBQ* mRNA levels and presented as the mean ± SE ($n = 3$). *$p$ <

0.01 compared to the same tissue in the other cultivar: blue asterisk – gene expression level in cv. Sarmat is higher than that in cv. Strelets; red asterisk – gene expression level in cv. Strelets is higher than that in cv. Sarmat.
